# Hernie hiatale par glissement du nourrisson

**DOI:** 10.11604/pamj.2013.15.78.2904

**Published:** 2013-06-26

**Authors:** Aliou Amadou Dia

**Affiliations:** 1 Service de radiologie de l'hôpital Saint-Jean de Dieu (Thiès), Sénégal

**Keywords:** Hernie hiatale, hiatus œsophagien, vomissements incoercibles, TOGD, hiatal hernia, esophageal hiatus, hyperemesis, UGI

## Images in médecine

La hernie hiatale du nourrisson par glissement est peu fréquente et correspond à une ascension de l'estomac dans le thorax à travers le hiatus œsophagien, l'angle cardio-tubérositaire se retrouvant en situation intra-thoracique. La hernie hiatale peut être asymptomatique ou se manifester par des symptômes chroniques non spécifiques comme tels que des vomissements intermittents attribués à un reflux gastro-œsophagien, des infections respiratoires récurrentes. Le mode de présentation peut également être aigu ou subaigu par des complications: un malaise grave, un syndrome occlusif le plus souvent incomplet, un volvulus gastrique intra-thoracique ou la migration intra-thoracique d'autres organes tels que le côlon et la rate. La radiographie thoracique peut parfois être normale si la hernie est intermittente ou montrer une image aérique médiastinale postérieure avec parfois un niveau hydro-aérique. Le diagnostic est confirmé par un TOGD qui montre une opacification de la grosse tubérosité gastrique dans le médiastin postérieur avec visualisation le plus d'un reflux associé. La PH-métrie qui enregistre le pH du bas-œsophage sur une longue période permet de quantifier le reflux acide dans l’œsophage. Quant à l'endoscopie, elle permet de visualiser une œsophagite et de le classifier. Nous rapportons le cas d'un nourrisson de 06 mois admis pour vomissements incoercibles, post-prandiaux précoces chez qui la radiographie thoracique réalisée a permis de suspecter une hernie hiatale devant la présence d'une image hydro-aérique retro-cardiaque et confirmée par le TOGD. Le traitement en première intention, est médical associe les épaississants, les médicaments anti-acides, les régulateurs du péristaltisme et des moyens posturaux comme la position verticale conservée un moment après la tétée et la position inclinée tête en haut pendant le sommeil. L'indication d'un traitement chirurgical est posée mais différée, devant la taille de la hernie hiatale et surtout le risque de complications.

**Figure 1:**
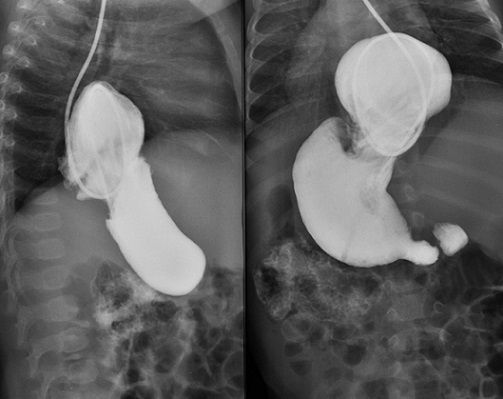
TOGD en incidence de profil et de face montrant une opacification du

